# Impact of WO_3_-Nanoparticles on Silicone Rubber for Radiation Protection Efficiency

**DOI:** 10.3390/ma15165706

**Published:** 2022-08-18

**Authors:** Hanan Al-Ghamdi, Hanaa M. Hemily, I. H. Saleh, Z. F. Ghataas, A. A. Abdel-Halim, M. I. Sayyed, Sabina Yasmin, Aljawhara H. Almuqrin, Mohamed Elsafi

**Affiliations:** 1 Department of Physics, College of Science, Princess Nourah bint Abdulrahman University, P.O. Box 84428, Riyadh 11671, Saudi Arabia; 2 Department of Environmental Studies, Institute of Graduate Studies and Research, Alexandria University, Alexandria 21526, Egypt; 3 Department of Basic and Applied Science, College of Engineering and Technology, The Arab Academy for Science, Technology and Maritime Transport, Alexandria 1029, Egypt; 4 Department of Physics, Faculty of Science, Isra University, Amman 11622, Jordan; 5 Department of Physics, Chittagong University of Engineering and Technology, Chattogram 4349, Bangladesh; 6 Physics Department, Faculty of Science, Alexandria University, Alexandria 21511, Egypt

**Keywords:** silicone rubber, WO_3_-nanoparticles, Cs-137, Co-60, Am-241

## Abstract

Silicone rubbers are a good choice for shielding materials because of having elastic and attenuating properties as well as cost-effectiveness. Thus, the aim of this study was to prepare ground-breaking silicone rubber samples by adding WO_3_-nanoparticles and testing the performance of their radiation shielding ability against Cs-137, Co-60, and Am-241 gamma energy. Increasing the concentration of WO_3_ nanoparticles in silicone rubber (SR) led to decreasing the half-value layer (HVL) and mean free path (MFP) values determined for the samples tested. Furthermore, the values of MFP and HVL upsurged according to the enhancement of the photon energy. It is noteworthy that the prepared silicone rubber (SR) systems with 50 and 60 wt% concentrations of WO_3_-nanoparticles displayed lower HVL than the Bi_2_O_3_-containing silicone rubber (SR) systems. In the same way, studied silicone rubber SR-W60 represented the lowest HVL comprising iron ore containing silicone rubber.

## 1. Introduction

As technology is improved and industries develop, there is an increased use in the number of machines that use radiation. Radiation-harnessing technologies are present in energy generation, medicine, agriculture, food processing, and more [1,2,3]. In the field of medicine, for example, radiation is used in the form of X-rays for medical imaging as well as in radiotherapy to eliminate cancer cells. As the benefits of ionizing radiation become more apparent, it is also necessary to consider the possible harmful effects of being exposed to high-energy photons. If the human body is exposed to large amounts of radiation, severe side effects can occur such as cancer development. To avoid all possible harmful effects of ionizing radiation, radiation shields are commonly placed between the source of high-energy photons and the human body [4,5,6,7].

Lead is the oldest and most widely known shielding material, in the form of lead aprons, for instance. Despite lead being an effective shield, it is naturally toxic to humans and the environment. Thus, it is preferable to find alternatives to minimize its use, especially in certain fields such as medicine, where some patients are more sensitive to lead toxicity [8,9,10,11,12].

Polymers are an alternative material to lead for radiation shielding applications. They are desirable shields due to their flexibility, low cost, lightweight, workability, chemical stability, etc. All of these properties make polymers effective absorbers of gamma rays. Previous studies have investigated adding PbO and other filler materials such as zinc borate to determine the potential of these rubbers in radiation shielding applications [13,14,15].

To improve the shielding ability of the rubbers, additives can be introduced into the polymers, such as bismuth, tungsten, and antimony powder. These additives increase the probability of the shield interacting with the incoming photons, enhancing the amount of radiation that is attenuated [16,17,18]. For example, Belgin et al. prepared a low-density polymer with PbO and WO_3_ and found that the polymer exhibits highly desirable characteristics when shielding against gamma rays [19]. Hassan et al. fabricated an epoxy matrix adding 30% weight of tungsten borides WB and WB_2_ microparticles for the purpose of radiation shielding. Attaining results showed that the combination of WB and WB_2_ microparticles to the epoxy matrix amended the gamma-ray shielding ability, and, considering gamma radiation shielding ability, the EP30WB_2_ micro composite showed higher attenuation results than the EP30WB composite [20]. Hashemi et al. displayed that the radio-opacity of GO-Pb_3_O_4_ containing epoxy increased with the increase in containing filler as well as its thicknesses [21]. Nazlıcan et al. fabricated an innovative, non-toxic, and low-cost epoxy polymer matrix with Yahyali Stone (YS) natural stone powder for gamma-ray shielding applications. Fe_2_O_3_ (75.28 wt%), SiO_2_ (17.21 wt%), and Al_2_O_3_ (4.24 wt%) are the components of the Yahyali Stone (YS) natural stone. Obtained results indicated that Epoxy/YS composites shielding capacity rises according to the enhancement of YS’s amount specifically consisting of Fe_2_O_3_. The Epoxy/YS composites are good for a low energetic gamma-ray utilization field such as nuclear medicine [22]. Nanomaterials have also been shown to provide an additional improvement over conventional micromaterials, as a smaller particle size typically correlates with greater attenuation. For example, Mahmoud et al. prepared high-density polymer composites with lead oxide nanoparticles, comparing them with using traditional bulk lead [23]. Tungsten specifically has higher attenuation coefficients than other common additives, making it a good powder to introduce to polymers.

Thus, this study will investigate the radiation shielding properties of silicone rubber with nano-WO_3_ to determine its viability in radiation shielding applications.

## 2. Materials and Method

**Figure 1:**
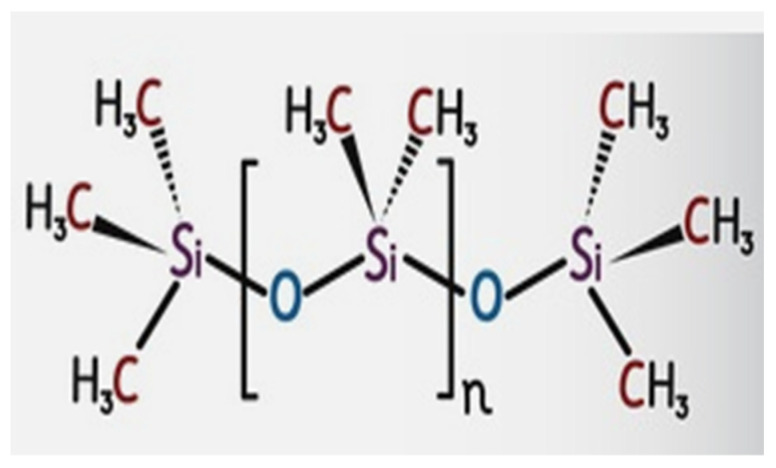
The chemical structure of silicone rubber.

**Figure 2:**
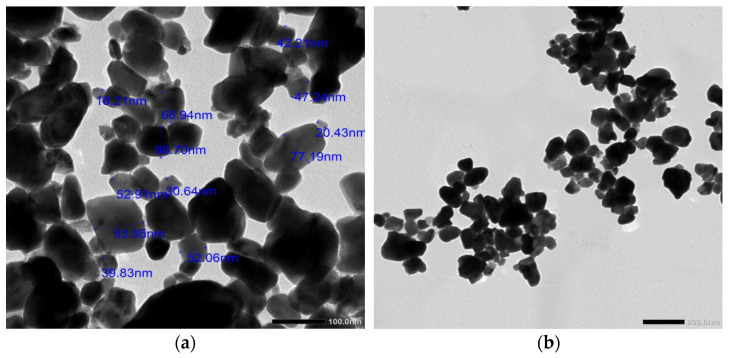
TEM image of WO_3_ nanoparticles (**a**) 100 nm scale and (**b**) 200 nm scale.

**Figure 3:**
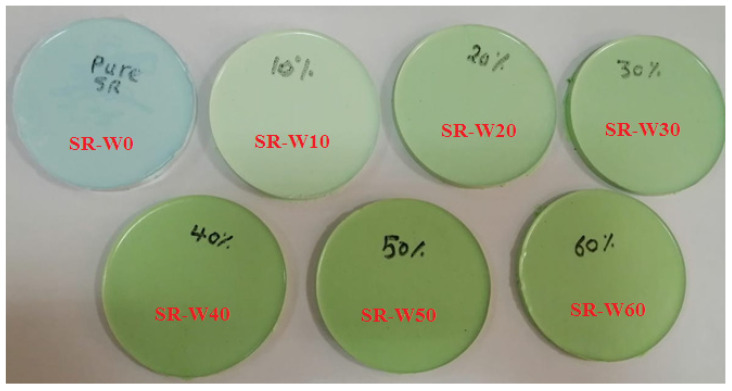
The prepared samples in this study.

New flexible samples were prepared to test their shielding ability. The samples were a mixture of silicone rubber (with a hardener) and tungsten oxide. Liquid poly(dimethylsiloxane) with chemical structure shown in Figure 1 was purchased and has the same properties that were previously studied [24,25,26]. Tungsten oxide has all its particles in the nano size, where the average particle size was 40 nm from TEM results. The TEM (transmission electron microscope) was applied for some powder WO_3_ nanoparticles as shown in Figure 2. Silicone rubber was mixed with tungsten oxide in proportions as shown in Table 1 in the traditional way, where the mixture was placed in a bowl and an electric mixer was used for 10 min until it became homogenous, so that the samples were stirred well and the samples were placed in cylindrical molds with a diameter of 8 cm. After preparation, the samples were left to dry for 24 h until the samples became flexible, as shown in Figure 3.

The mechanical and morphological properties were studied with the same devices taken from the literature data [24], where the tensile strength and Young’s modulus were evaluated for the prepared SR-WO_3_ samples using an electronic tensile testing machine (model 1425, Germany), according to standard techniques with ASTM D412 as well as a scanning electron microscope (SEM2) of SEM-T200, JEOT model (Akishima, Japan), which was used to scan the prepared samples under operating voltage 20 keV and magnification number around 35,000.

**Figure 4:**
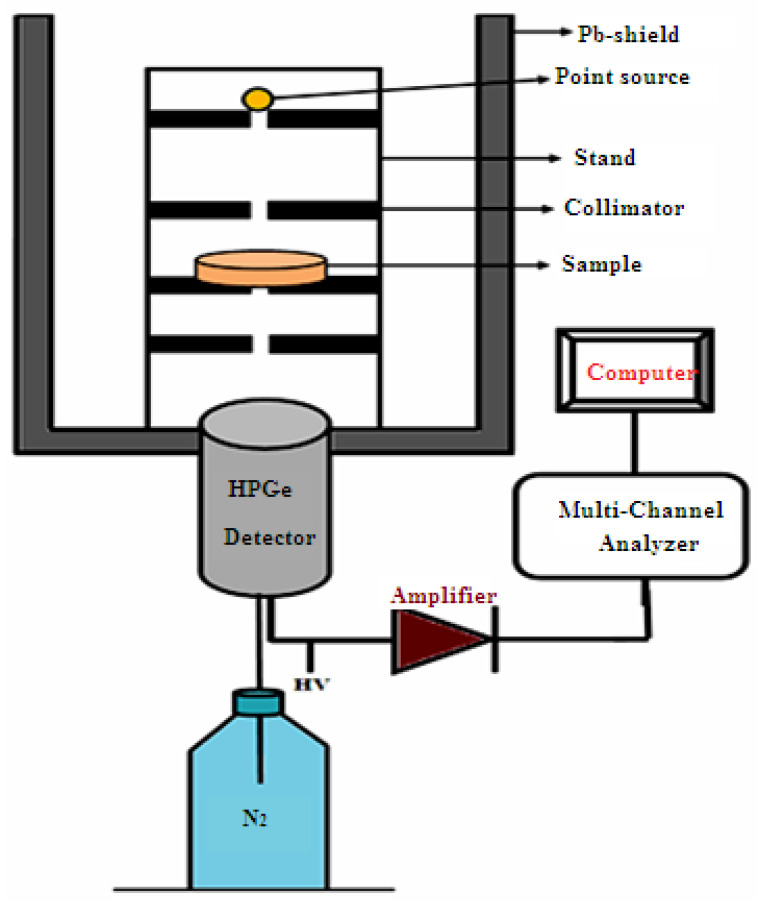
The illustration setup of the experimental work.

The samples were exposed to three radioactive sources (Cs-137, Co-60, Am-241) and the intensity of incident radiation in the presence (I) and absence of the sample (I_0_) were measured using HPGe detector [27,28]. The sample is placed between the radioactive source and the detector at a suitable point as shown in Figure 4. The sample was measured for a different thickness from the same sample with the fixed measurement time. By determining the intensity in both cases (the presence and absence of silicone rubber) and calculating the thickness of the sample and by applying the Lambert-Beer’s law, the linear attenuation coefficient (LAC) was determined by the following equation [29,30].

(1) $$LAC=\frac{1}{x}\ln\;\frac{I_{0}}{I}$$

where ‘*x*’ represents the thickness of the silicone rubber sample. HVL and MFP are important parameters describing the thickness of the sample in which the radiation intensity is halved and the distance of radiation travels within that thickness without any interaction, respectively, and given by the following equations [31].

(2) $$HVL=\frac{\mathrm{Ln}\left(2\right)}{LAC}\;\;\;,\;\;\;\;\;\;\;MFP=\frac{1}{LAC}$$

## 3. Results and Discussion

**Figure 5:**
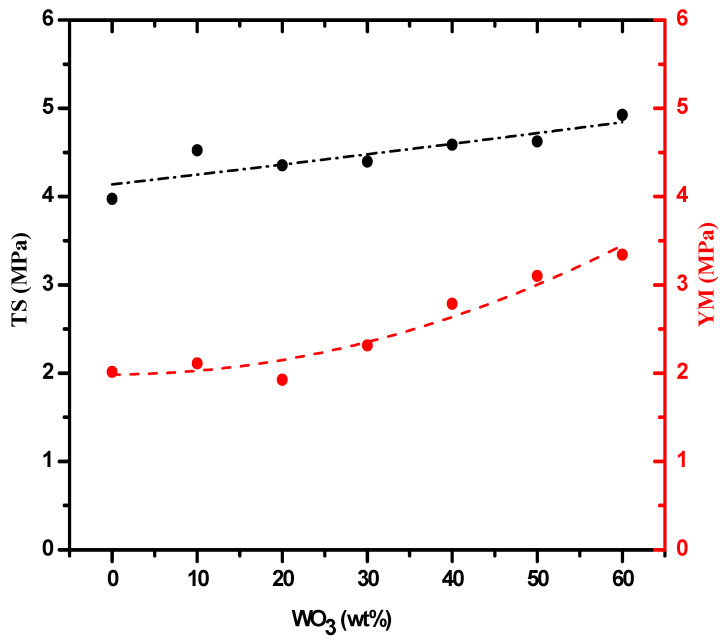
The tensile strength (TS) and young modulus of the SR-WO_3_ systems.

The tensile strength (TS, MPa) of the prepared samples was calculated in addition to Young’s modulus (YM, MPa). It was clear from the results as shown in Figure 5 that adding WO_3_ nanoparticles positively affected the tensile strength and Young’s modulus, where the tensile strength of silicone rubber ranged from 3.975 MPa in the absence of additives (SR-W0), while it was 4.295 when 60% of nanoparticles was added (SR-W60). Similarly, the results of Young’s modulus were positive with the increase in WO_3_ nanoparticles as shown in the figure, and this was reported in other papers, indicating that the tungsten nanoparticles positively affect the mechanical properties when added to the polymer in general [32,33].

**Figure 6:**
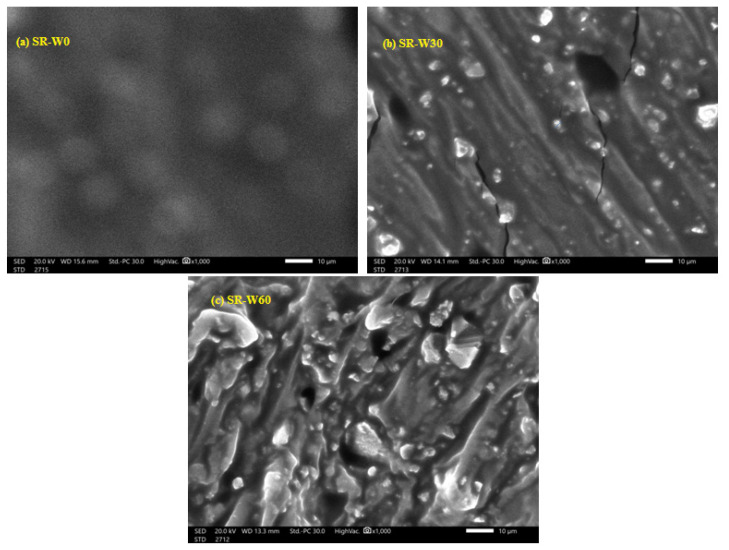
SEM images of (**a**) SR-W0, (**b**) SR-W30 and (**c**) SR-W60.

A scanning electron microscope (SEM-IT 200) was used to scan the prepared samples, and it was found that the nanoparticles kept their size inside the silicone rubber, but with an increase in the filler percentage, some aggregates of nanoparticles were found inside the polymer. In Figure 6, the nanoparticles were clear without any aggregates when mixed with 30% WO_3_ (SR-W30), while during scanning the sample with the highest percentage of (SR-W60), some aggregates were found, but it did not affect its mechanical and attenuation properties, as shown below.

**Figure 7:**
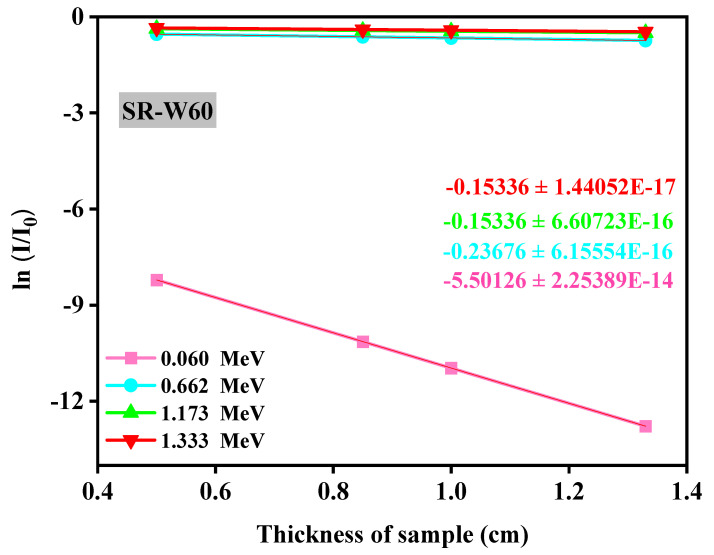
Diagrammatic presentation of the fractional transmission (ln(I/I_0_)) versus thickness of prepared silicone rubber sample SR-W60 system.

**Figure 8:**
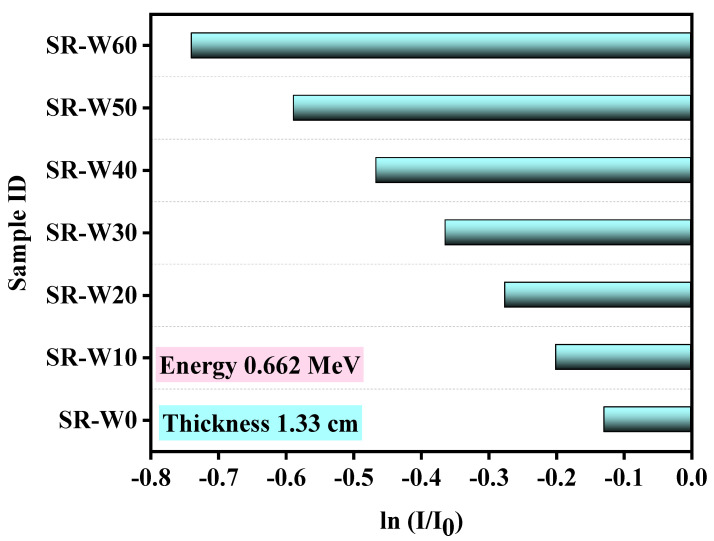
Diagrammatic presentation of the fractional transmission (ln(I/I_0_)) versus WO_3_ concentration on prepared silicone rubber system for thickness of 1.33 cm at energy 0.662 MeV.

The fractional transmission (ln(I/I_0_)) versus the thickness (cm) of silicone rubber (SR) systems with different concentrations of WO_3_-nanoparticles for energy 0.0595, 0.662, 1.173, and 1.333 MeV have been publicized. The slope representing the most fitting straight line considering these data are of utmost importance as LAC has been displayed through the slope of the line obtained after scheming the fractional transition values ln(I/I_0_) versus thickness of the absorbing materials (considering Lambert_Beer’s law). Herein, an increase in the energy causes an increase in the I/I_0_ and this means that the photons with high energy can penetrate the prepared silicone rubber easier than the photons with low energy. The negative value of the slope revealed that the transmitting value declined according to the enhancement of the absorbers’ thickness. A typical figure (Figure 7) has been presented here with the most fitting straight-line view with the slope value of 5.50126 denoting the linear attenuation coefficient (cm^−1^) at 0.06 MeV. It is most convenient to attain mass attenuation coefficient (MAC) through the normalization of LAC by the density of that material. MAC has been found to be 2.33 gm/cm^2^ through normalizing the LAC (5.50126 cm^−1^) by the density (2.37 gm/cm^3^) of the prepared silicone rubber (SR-W60), and it is notable that the I/I_0_ has an inverse relationship, meaning I/I_0_ decreases with increasing the thickness of the absorbing material. Thus, it is better to prepare a sample with high thickness in order to get materials with good shielding properties. Hence, these prepared silicone rubber (SR-W60) samples represented in this work showed that silicone rubber (SR-W60) with the highest density (2.37 gm/cm^3^) displayed the superior MAC vale. It is clear from Figure 8. that with the increase in WO_3_ concentration on the prepared silicone rubber (SR) hence the value of fractional transition ln(I/I_0_) decreases, which demonstrates that the enhancement of the amount of WO_3_ boosts the attenuation performance of the prepared silicone rubber (SR) samples.

**Figure 9:**
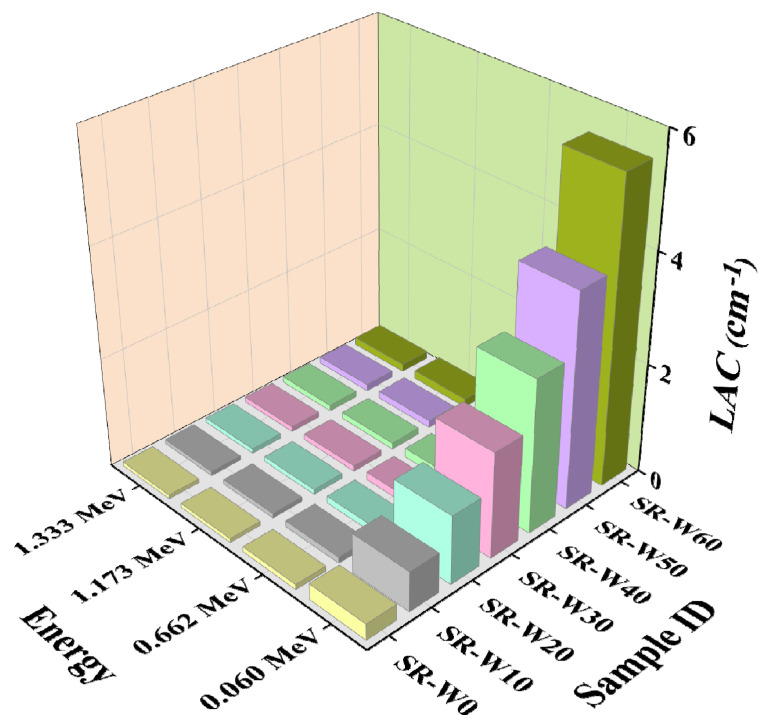
Pictorial presentation of the LAC of prepared silicone rubber (SR-W) systems with the function of energy.

**Figure 10:**
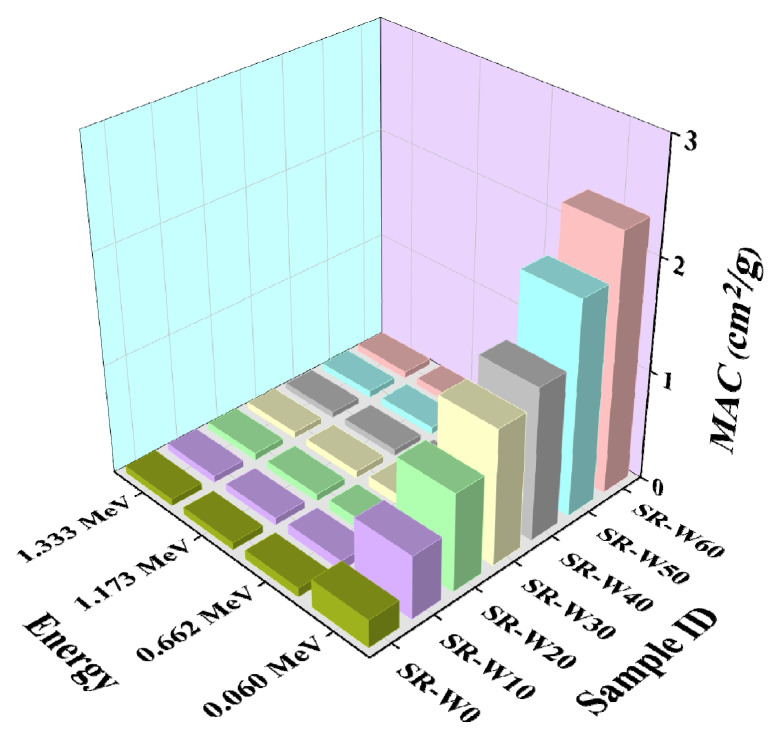
Pictorial presentation of the MAC of prepared silicone rubber (SR-W) systems with the function of energy.

**Figure 11:**
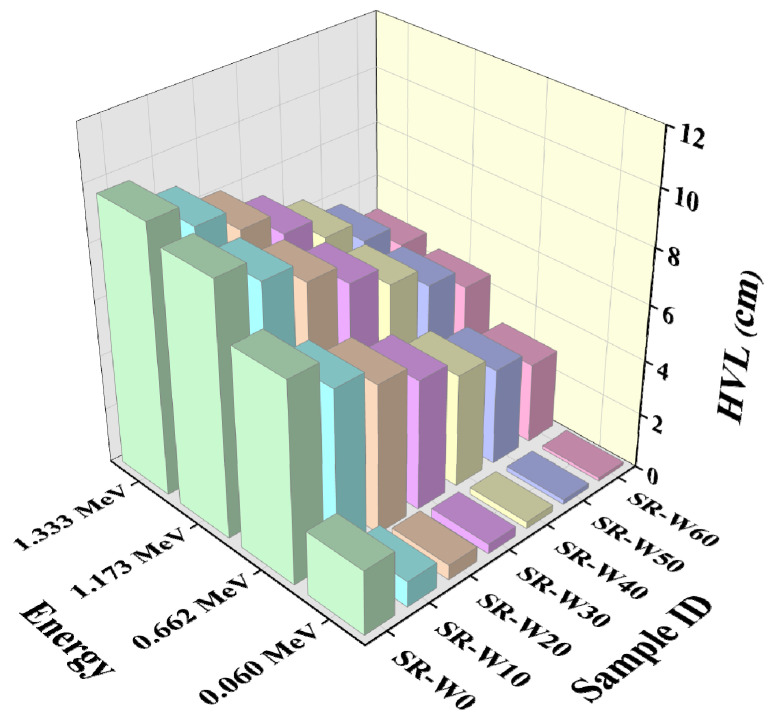
Pictorial presentation of the HVL of prepared silicone rubber (SR-W) systems with the function of energy.

**Figure 12:**
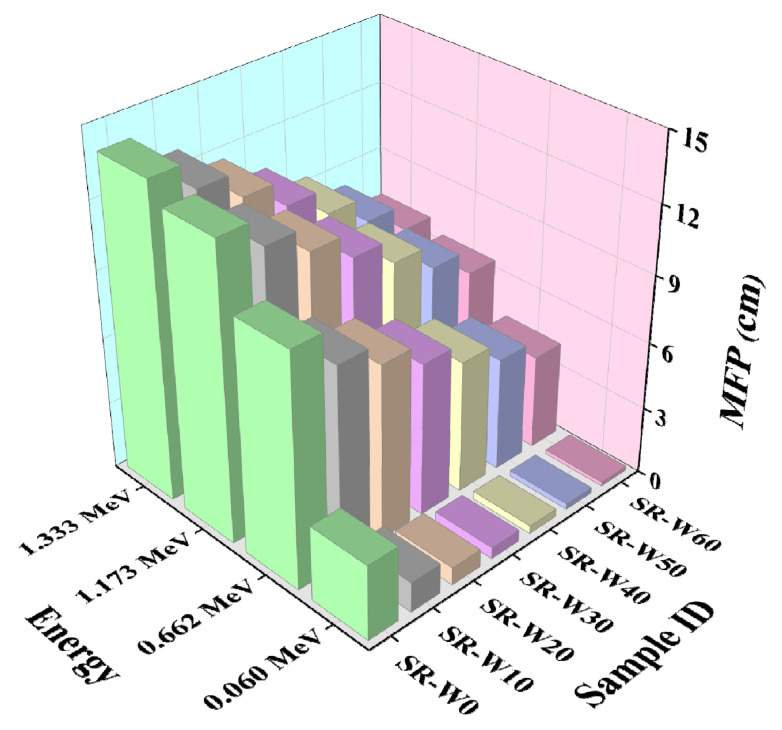
Pictorial presentation of the MFP of prepared silicone rubber (SR-W) systems with the function of energy.

In this study, prepared silicone rubber (SR) containing 0, 5, 10, 30, 40, 50, and 60 wt% concentrations of WO_3_-nanoparticles have been examined to get the values of linear attenuation coefficient (LAC), mass attenuation coefficient (MAC), half-value layer (HVL), and mean free path (MFP). Depicted figures (in Figure 9, Figure 10, Figure 11 and Figure 12) assessed the values of LAC, MAC, HVL, and MFP of the prepared silicone rubber (SR) dependent upon the photon energies, correspondingly. Prepared silicone rubber (SR-W50) has shown that the value of LAC (0.13 cm^−1^) at the energy of 1.173 MeV whereas the value of LAC (3.9 cm^−1^) at the energy of 0.060 MeV which is 31 times higher (Figure 9). This is a clear clarification of the effect of the energy on the LAC for the prepared silicone rubber systems. Increasing the concentration of WO_3_ nanoparticles in silicone rubber (SR) led to maintaining the downward direction of SR-W0 > SR-W10 > SR-W20 > SR-W30 > SR-W40> SR-W50 > SR-W60 for the obtained values of the HVL and MFP of the current study.

These results show that the radiation shielding ability of any substance has an inverse dependence on the density of the prepared silicone rubber (SR). Consequently, the highest density holding silicone rubber (SR-W60) has provided the maximum shielding competence against gamma photons compared to the rest of the prepared silicone rubber (SR) systems which specify the effect of WO_3_ content or the density on the LAC. Obtained figures show that the values of MFP and HVL v upsurge according to the enhancement of the photon energy.

**Figure 13:**
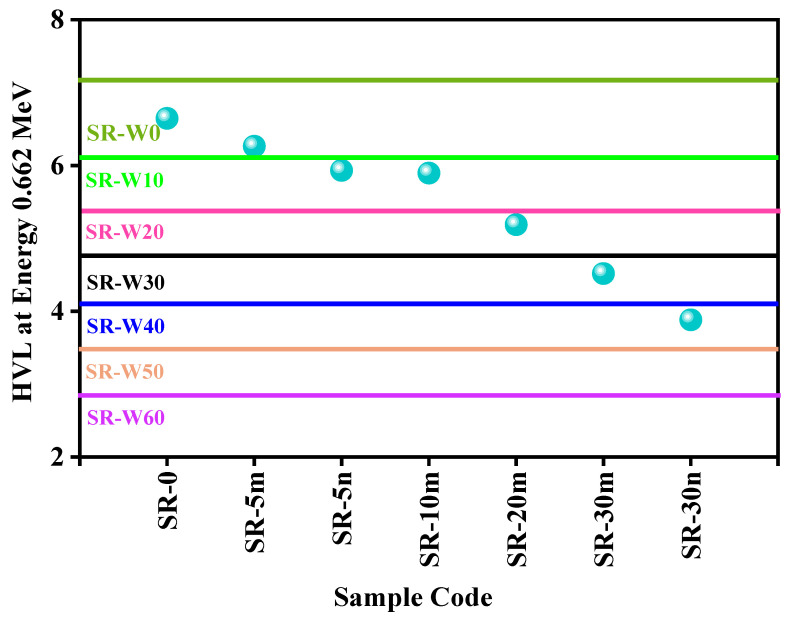
The comparison of HVL between the WO_3_-containing silicone rubber (SR-W0) systems with Bi_2_O_3_-containing silicone rubber (SR-0) systems at energy 0.662 MeV.

Figure 13 represents the comparison of HVL between the different concentrations of WO_3_-containing silicone rubber (SR-W0) system with the numerous concentrations of Bi_2_O_3_-containing silicone rubber (SR-0) systems [24] at energy 0.662 MeV. In Figure 13, the symbol indicates that SR-5m (micro-sized Bi_2_O_3_ particle) and SR-5n (nano-sized Bi_2_O_3_ particle), etc.

**Figure 14:**
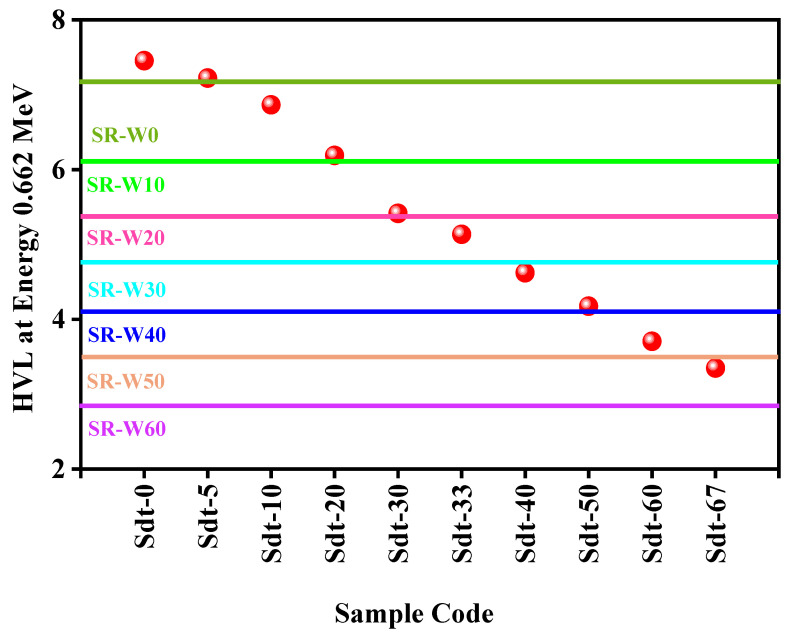
The comparison of HVL between the WO_3_-containing silicone rubber (SR-W0) system with iron ore-containing silicone rubber (Sdt-0) systems at energy 0.662 MeV.

0, 10, 20, 30, 40, 50, and 60 wt% of WO_3_-nanoparticles contaminated silicone rubber (SR) systems (present study) have been compared to the silicone rubber (SR) systems with 0, 5, 10, 30, and 30 wt% of micro and nano sizes Bi_2_O_3_ (literature data) fabricated by Abbas et al. [24]. In Figure 13, the blue colored spheres indicate the HVL values of the compared samples (taken from the literature data [24]) at 0.0662 MeV. This figure displays that SR-W50 and SR-W60 have lower HVL than the Bi_2_O_3_-containing silicone rubber (SR) systems. The comparison of HVL between the WO_3_-containing silicone rubber (SR-W0) system with iron ore-containing silicone rubber (Sdt-0) systems [26] has been presented in Figure 14 for energy 0.662 MeV. In Figure 14, red colored balls indicate the value of compared samples’ (literature data—[26]) HVL at energy 0.0662 MeV. Silicone rubber SR-W60 (prepared sample) has represented the lowest HVL comprising pure and up to 67 wt% of iron ore added to silicone rubber (literature data—[26]).

## 4. Conclusions

Silicone rubber samples have been prepared by adding 0, 10, 20, 30, 40, 50, and 60 wt% of WO_3_-nanoparticles. The attained values of linear attenuation coefficient (LAC), mass attenuation coefficient (MAC), half-value layer (HVL), and mean free path (MFP) showed that enhancing the number of WO_3_-nanoparticles on silicone rubber boosts its attenuation efficiency against gamma rays. The highest density holding silicone rubber (SR-W60) provided the maximum shielding competence against gamma photons compared to the rest of the prepared silicone rubber (SR) systems Moreover, the values of MFP and HVL upsurged according to the enhancement of the photon energy. It is notable that at energy 0.0662 MeV, the prepared silicone rubber samples SR-W50 and SR-W60 displayed lower HVL than the Bi_2_O_3_-containing silicone rubber (SR) systems. In the same way, studied silicone rubber SR-W60 represented the lowest HVL compared with silicone rubber containing 67% iron particles at 0.0662 MeV.

## Figures and Tables

**Table 1:** Chemical composition, densities, and the codes of the prepared flexible samples.

Codes	Chemical Compositions (wt%)		Density (g/cm^3^)
	Silicone Rubber	WO_3_-Nanoparticles	
SR-W0	100	0	1.180 ± 0.008
SR-W10	90	10	1.293 ± 0.011
SR-W20	80	20	1.421 ± 0.008
SR-W30	70	30	1.580 ± 0.009
SR-W40	60	40	1.779 ± 0.007
SR-W50	50	50	2.032 ± 0.008
SR-W60	40	60	2.375 ± 0.009

## Data Availability

Not applicable.
